# Metabolic Plasticity in Resting and Thrombin Activated Platelets

**DOI:** 10.1371/journal.pone.0123597

**Published:** 2015-04-13

**Authors:** Saranya Ravi, Balu Chacko, Hirotaka Sawada, Philip A. Kramer, Michelle S. Johnson, Gloria A. Benavides, Valerie O’Donnell, Marisa B. Marques, Victor M. Darley-Usmar

**Affiliations:** 1 Department of Pathology, University of Alabama at Birmingham, Birmingham, Alabama, United States of America; 2 UAB Mitochondrial Medicine Laboratory, University of Alabama at Birmingham, Birmingham, Alabama, United States of America; 3 Center for Free Radical Biology, University of Alabama at Birmingham, Birmingham, Alabama, United States of America; 4 Department of Medical Biochemistry, Cardiff University, Cardiff, United Kingdom; University of South Alabama, UNITED STATES

## Abstract

Platelet thrombus formation includes several integrated processes involving aggregation, secretion of granules, release of arachidonic acid and clot retraction, but it is not clear which metabolic fuels are required to support these events. We hypothesized that there is flexibility in the fuels that can be utilized to serve the energetic and metabolic needs for resting and thrombin-dependent platelet aggregation. Using platelets from healthy human donors, we found that there was a rapid thrombin-dependent increase in oxidative phosphorylation which required both glutamine and fatty acids but not glucose. Inhibition of fatty acid oxidation or glutamine utilization could be compensated for by increased glycolytic flux. No evidence for significant mitochondrial dysfunction was found, and ATP/ADP ratios were maintained following the addition of thrombin, indicating the presence of functional and active mitochondrial oxidative phosphorylation during the early stages of aggregation. Interestingly, inhibition of fatty acid oxidation and glutaminolysis alone or in combination is not sufficient to prevent platelet aggregation, due to compensation from glycolysis, whereas inhibitors of glycolysis inhibited aggregation approximately 50%. The combined effects of inhibitors of glycolysis and oxidative phosphorylation were synergistic in the inhibition of platelet aggregation. In summary, both glycolysis and oxidative phosphorylation contribute to platelet metabolism in the resting and activated state, with fatty acid oxidation and to a smaller extent glutaminolysis contributing to the increased energy demand.

## Introduction

Platelets are circulating cytoplasmic fragments of megakaryocytes, which reside in the bone marrow. Platelets do not have nuclei, but contain a number of organelles such as mitochondria, lysosomes and peroxisomes [1]. The primary role of platelets is to mediate hemostasis through thrombus formation. Thrombin is a pro-coagulant factor that is produced during the coagulation cascade and stimulates platelets to change their shape, adhere to the endothelium, aggregate, release the contents of dense and alpha granules and mediate clot retraction, all of which are energetically demanding processes [2–4]. The understanding of the metabolic changes required for activation and aggregation of platelets is paramount in trying to design effective interventions to target diseases of platelet dysfunction in both hyper and hypo-thrombotic events.

Both mitochondrial oxidative phosphorylation and glycolysis are highly active in platelets [5]. It has been estimated that in the resting platelet, 65% of the ATP is generated from glycolysis and 35% from oxidative phosphorylation [6]. As expected, the rate of glycolysis increases as the oxygen tension decreases [7]. On stimulation of platelet aggregation both oxidative phosphorylation and glycolysis are engaged, but the substrates required for this process are unknown and the ability of the pathways to compensate for each other has not been investigated [6,8–10]. In some studies, components of the mitochondrial respiratory chain have been inhibited, and these studies concluded that mitochondrial function is essential to provide the ATP necessary for platelet aggregation [11–13]. However, other reports have stated that glycolysis is the major source of ATP in driving platelet aggregation, and that mitochondria play only a minor role [6,14,15]. Inhibition of both glycolysis and oxidative phosphorylation in concert completely abolishes platelet aggregation, which would indicate that both metabolic processes could be important [10]. This suggested to us that the platelet can exhibit metabolic plasticity in the substrates it uses for aggregation. Other studies have suggested that the mitochondrial permeability transition pore is opened during thrombin-dependent aggregation [16,17]. Since the opening of the pore depolarizes the mitochondrial inner-membrane, and so prevents the synthesis of ATP, this would be consistent with a pro-apoptotic signaling role for the organelle but precludes a contribution to platelet bioenergetics [18–20].

Mitochondrial fatty acid oxidation can contribute to ATP production in platelets in both the resting and thrombin stimulated state [21,22]. Platelets contain the necessary enzymes for *de novo* synthesis of fatty acids, and are also able to transport extracellular fatty acids for use as energetic substrates [23]. It has been shown that inhibition of fatty acid metabolism through inhibitors of carnitine palmitoyltransferase-1 (CPT-1), have no effect on platelet aggregation [24]. L-glutamine (Gln) is also an important substrate that fuels oxidative phosphorylation through its conversion to glutamate and then alpha-ketoglutarate, a substrate for the TCA cycle, in a process termed glutaminolysis, and is functional in platelets [25,26]. Importantly, the dynamic interaction between these metabolic pathways during thrombin-dependent aggregation has not been investigated.

In the present study, we utilized a state-of-the art bioenergetic analysis of intact platelets, to measure the role of glucose, mitochondrial fatty acid oxidation and Gln in supporting metabolism, and determined the substrate’s ability to meet the energetic demand associated with thrombin-dependent aggregation. We confirmed that thrombin stimulates glycolysis and mitochondrial oxygen consumption, but for the first time demonstrate that oxidative phosphorylation, which is engaged rapidly on thrombin stimulation, is partly dependent on Gln availability and the ability of the mitochondria to oxidize fatty acids. Overall, our data show an integrated energetic response between both glycolysis and oxidative phosphorylation, with a stimulation of both ATP linked respiration and utilization of the bioenergetic reserve capacity. Inhibition of both pathways results in the synergistic inhibition of platelet aggregation. While, both fatty acids and Gln support oxidative phosphorylation for resting and thrombin stimulated platelets, when inhibited alone or in combination did not affect aggregation, consistent with the ability of glycolysis to compensate for loss of either fuel. These data reveal the metabolic plasticity of the platelet in both the resting and activated state.

## Materials and Methods

All reagents were purchased from Sigma-Aldrich (St. Louis, MO, USA) unless otherwise specified.

### Platelet Isolation

Platelet concentrates from individual donors was obtained for each experiment from The University of Alabama at Birmingham blood bank. Collection and use of these samples was approved by the University of Alabama at Birmingham Institutional Review Board (Protocol #X110718014). The protocol for isolating platelets from blood draws from healthy individuals was also approved by the University of Alabama at Birmingham Institutional Review Board. Written informed consent was obtained from each donor and documented as approved by the University of Alabama at Birmingham Institutional Review Board (Protocol #X110718014). Blood draws from healthy volunteers were obtained from males and females between the ages of 25–60 who have no chronic diseases. Platelets used for these studies were between day 6 and 8 after collection or freshly isolated as described previously [27,28]. In brief, platelets were pelleted by centrifuging at 1500 g for 10 minutes then washed with PBS containing prostaglandin I_2_ (1 μg/ml) and platelet number was determined by turbidimetry [29]. The platelets for the experiments were isolated from 9 individual donors, and we noted some individual variation in the range of basal oxygen consumption rate (OCR) from 8–15 pmol/min/μg protein and the extracellular acidification rate (ECAR) measurements ranged from 3–6 mpH/min/μg protein. The response to thrombin (0.5 Units/ml (U/ml)) was determined and compared to freshly isolated platelets and no significant differences were noted in the stimulation of mitochondrial function or glycolysis. There was also no cytotoxicity associated with thrombin treatment (0.5 U/ml) over the time period of these experiments, as assessed by release of the intracellular enzyme lactate dehydrogenase.

### Assessment of platelet bioenergetics and aggregation

The 96-well format Seahorse extracellular flux analyzer (Seahorse Bioscience, MA, USA) was used to measure bioenergetics [27]. Platelets were diluted to a concentration of 1 x 10^7^ in XF DMEM assay buffer (DMEM with 1 mM pyruvate, 5.5 mM D-glucose, 4 mM L-glutamine, pH 7.4) and were seeded onto Cell-Tak coated XF96 microplates and mitochondrial stress test was performed as described [30]. In these experiments thrombin (0.5 U/ml) was used to stimulate platelet aggregation. These assays were also performed with a 1 h pre-treatment of platelets with BSA-palmitate (200 μM), etomoxir (25 μM), 2-deoxy-D-glucose (2DG, 120 mM), koningic acid (10 μM), XF assay medium with or without Gln or 3h pre-treatment with trimetazidine (TMZ, 0–1000 μM), azaserine (Aza, 0–50 μM). BSA was conjugated to palmitate and the assay was performed with carnitine (500 μM) supplemented XF DMEM media as described previously [31]. Platelet aggregation using the 96 well plate reader was measured as previously described [32]. Aggregation data is shown as the percent change in transmittance compared to thrombin. The mitochondrial complex assay is performed using saponin (60 μg/ml) to permeabilize the plasma membrane as described [33].

### Measurement of NAD, AMP, ADP and ATP

Platelets (1 x 10^8^) in 0.75 ml of Seahorse media were seeded onto Cell-Tak coated 48-well plates. Next, the platelets were treated with either vehicle or thrombin (0.5 U/ml) and allowed to incubate for 30 min. Nucleotide analysis was performed using a modified method [34,35]. In brief, following treatment, 5% perchloric acid was added and platelets were harvested. This lysate was centrifuged and the supernatant was neutralized with K_2_HPO_4_ and stored at -80°C until HPLC analysis. The HPLC system consisted of a Gold HPLC model equipped with System Gold 168 Detector and System Gold Autosampler 507 from Beckman Coulter. The analytical column was a Supelcosil LC-18-T, (150 x 4.6 mm ID, particle size 3 μm) from Sigma. Analytical runs were processed by 32 Karat Software (version 8.0) also from Beckman Coulter Inc.

### Statistical analysis

Each experiment reported in this study is a representative of 2–3 experiments with platelets derived from different donors. Each platelet group is comprised of 3–5 technical replicates, and the data is presented as mean ± SEM. Statistical significance was determined using a either a t-test or ANOVA with Tukey post-hoc test for data with more than 2 groups, and p<0.05 was considered significant.

## Results

### The effect of thrombin on oxidative phosphorylation and glycolysis

**Fig 1:**
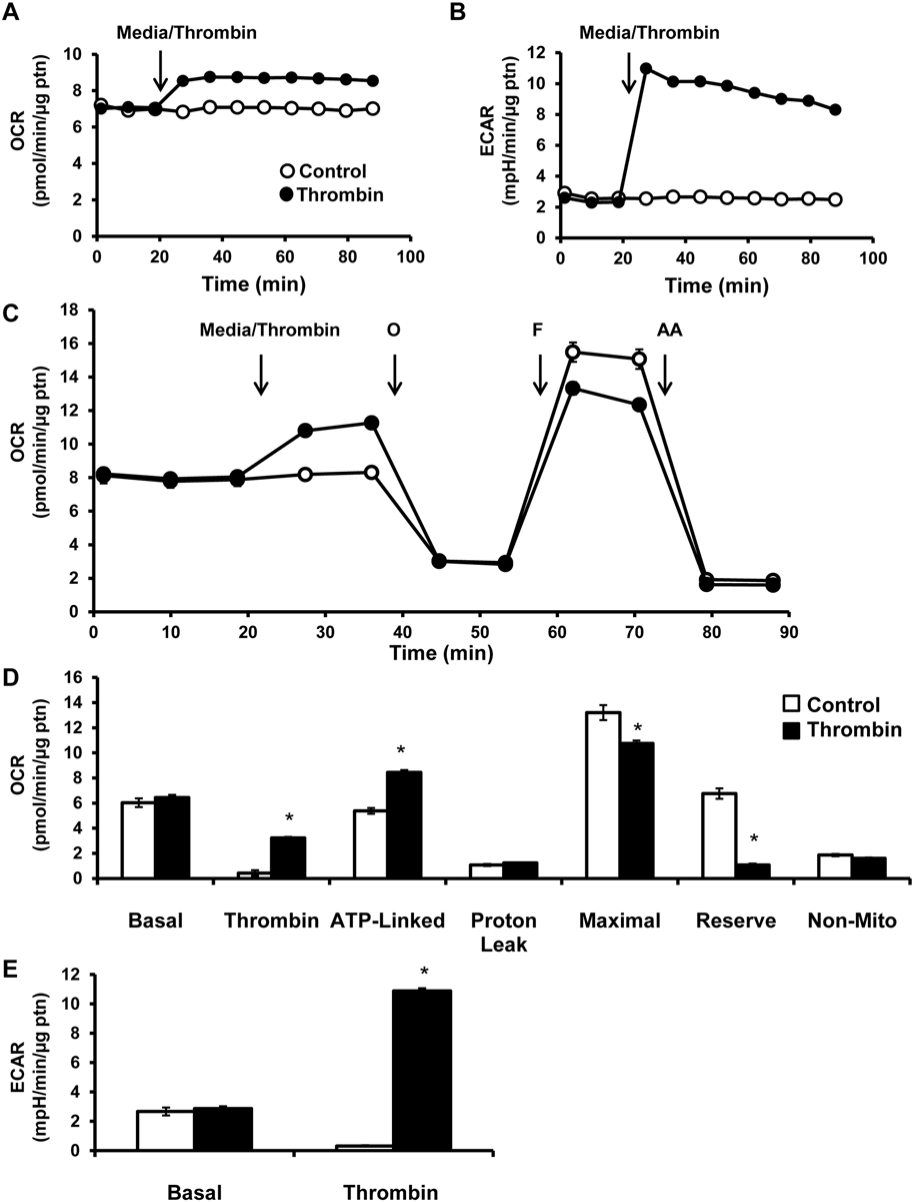
Bioenergetic profile of platelets exposed to thrombin. (A) OCR and (B) ECAR were measured in platelets by establishing a basal rate then injecting thrombin (0.5 U/ml) and following over 64 min. (C) either media or thrombin were injected (0.5 U/ml) and then sequential injections of 1 μg/ml oligomycin (O), 0.6 μM FCCP (F) and 10 μM Antimycin A (AA). (D) Different parameters of mitochondrial function were calculated—basal (basal OCR—AA sensitive OCR), thrombin (thrombin response—basal OCR), ATP-linked (thrombin response—oligomycin sensitive OCR), proton leak (oligomycin sensitive—AA sensitive OCR), maximal (FCCP sensitive—AA sensitive OCR), reserve capacity (FCCP sensitive—thrombin responsive OCR) and non-mitochondrial (AA sensitive OCR). (E) Simultaneously ECAR measured by first establishing a basal rate followed by injection of thrombin (0.5 U/ml). All the bioenergetic measurements were normalized to protein content per well. Data expressed as mean±SEM from one representative donor, n = 3–5 replicates per sample. *p<0.01, different from control.

Basal oxygen consumption rate (OCR) and extracellular acidification rate (ECAR) in platelets were established after which either media alone or thrombin (0.5 U/ml) was injected and followed for 64 min. Injection of thrombin stimulated OCR approximately 25% and this was sustained over the next hour (Fig 1A). Similarly, ECAR increased from 3 mpH/min/μg protein to 10 mpH/min/μg protein over the same time period (Fig 1B).

A mitochondrial stress test was performed to elucidate the effect of thrombin on mitochondrial function. First a basal rate of OCR was established, after which either media or thrombin (0.5 U/ml) was injected and followed for 16 min (Fig 1C). Injection of thrombin showed an increase in OCR which reached a stable rate after 8 min (Fig 1C and 1D). After 16 min, the complex V inhibitor, oligomycin (1 μg/ml) was injected and the anticipated decrease in OCR was observed in both the control and thrombin group. In order to estimate the maximal OCR, the proton ionophore, FCCP (0.6 μM) was added, which resulted in stimulation of respiration in the control group and to a lesser extent in the thrombin group (Fig 1C and 1D). To measure non-mitochondrial sources of oxygen consumption, the mitochondrial complex III inhibitor, antimycin A (10 μM), was injected 16 min after FCCP, which caused a decrease in OCR in both groups (Fig 1C and 1D). To calculate thrombin stimulated respiration, the rate of OCR after thrombin was subtracted from basal (Fig 1D). Based on the injection of oligomycin to inhibit ATP synthase, the indices of ATP linked respiration and proton leak can be calculated. ATP linked respiration was calculated by subtracting the rate of oxygen consumption after oligomycin addition from the basal rate of OCR. ATP linked respiration reflects oxygen consumption that is coupled to mitochondrial ATP production and increased in response to thrombin injection (Fig 1D). Proton leak was calculated by subtracting the rate of oxygen consumption after antimycin A injection from the rate of oxygen consumption after oligomycin addition. Proton leak is a measure of oxygen consumption that is not linked to ATP production and is an indicator of uncoupling, and was not changed in response to thrombin (Fig 1D). Bioenergetic reserve capacity was calculated by subtracting basal oxygen consumption from the rate of oxygen consumption after FCCP injection. Thrombin caused a decrease in reserve capacity of 84% compared to the control group (Fig 1D) in this platelet donor. We observed that in some cases reserve capacity could also be negative in the presence of thrombin. It is important to note that since non-mitochondrial respiration did not change significantly in response to thrombin, we can then conclude that the thrombin-dependent increase in OCR is mitochondrial in origin. Extracellular acidification (ECAR) was also measured in response to thrombin and found to be stimulated approximately 300% (Fig 1B and 1E).

### Effect of thrombin on complex I and II mediated respiration

**Fig 2:**
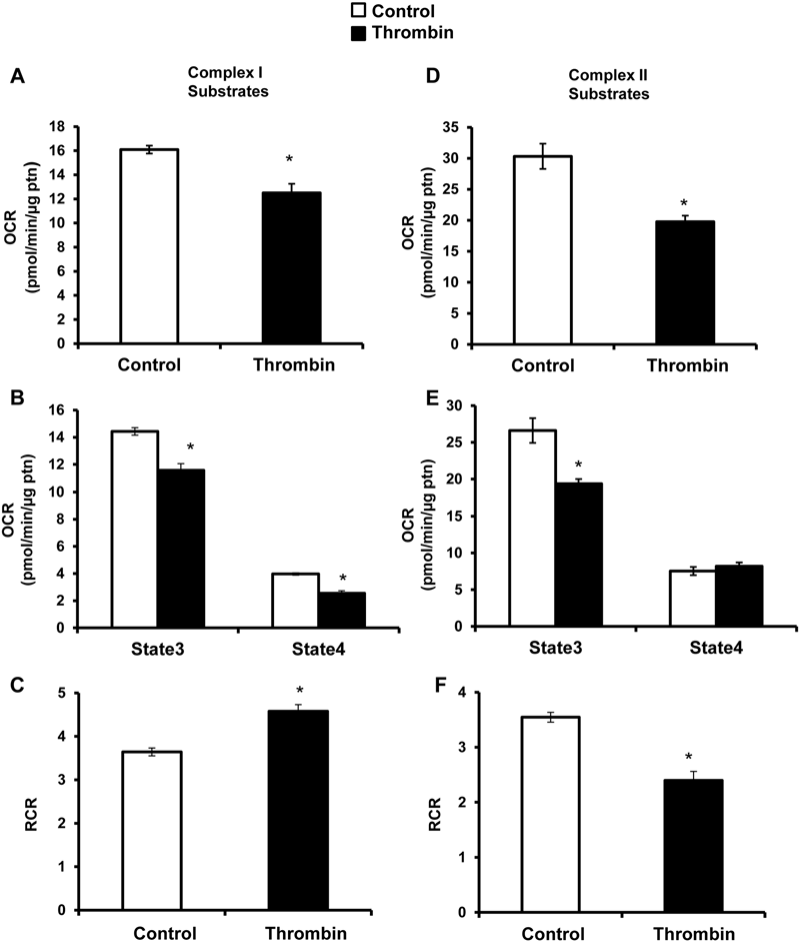
Mitochondrial assay of platelets exposed to thrombin. Platelets were plated on XF96 plates in MAS buffer, and first a basal rate of oxygen consumption was measured, followed by injection of thrombin (0.5 U/ml). This was followed by injection of (A) saponin (60 μg/ml), pyruvate (5 mM), malate (2.5 mM) and ADP (1 mM) for complex I substrates (D) or saponin (60 μg/ml), succinate (10 mM) and ADP (1 mM) for complex II substrates. Then oligomycin (1 μg/ml) and antimycin A (10 μM) were injected sequentially. From this different parameters of respiration were calculated—(B, E) state 3 (substrate sensitive—oligomycin sensitive OCR) and state 4 (oligomycin sensitive—AA sensitive OCR). (C, F) Respiratory control ratio (RCR) was calculated using the formulae—state3/state4. Data expressed as mean±SEM from one representative donor, n = 3–5 replicates per sample. *p<0.01, different from control.

The decrease in reserve capacity observed after the addition of thrombin could be explained by inhibition of the mitochondrial electron transport chain. To test this, the plasma membrane was permeabilized with saponin (60 μg/ml) to allow delivery of complex I and II substrates and ADP. Injection of pyruvate (5 mM) and malate (2.5 mM) which are complex I linked substrates) or succinate (10 mM, complex II linked substrates) together with ADP (1 mM) enabled the measurement of maximal (state 3) respiration (Fig 2A and 2D). Complex II linked substrate oxidation was greater than complex I, and both were inhibited 20–30% in response to thrombin (Fig 2A and 2D). Next, oligomycin (1 μg/ml) and antimycin A (10 μM) were injected, to allow measurement of state 4 respiration, and estimation of the respiratory control ratio (RCR) (Fig 2B, 2C, 2E and 2F). With 0.5 U/ml thrombin, a decrease in state 3 and 4 respiration for complex I substrates, and a decrease in state 3 only for complex II substrates was observed (Fig 2B and 2E). The RCR after thrombin increases with complex I substrates and decreases approximately 30% with complex II linked substrates (Fig 2C and 2F). Taken together these data are consistent with functional, well coupled mitochondria capable of generating ATP without any evidence of overt bioenergetic dysfunction.

### Effect of thrombin on nucleotides

**Fig 3:**
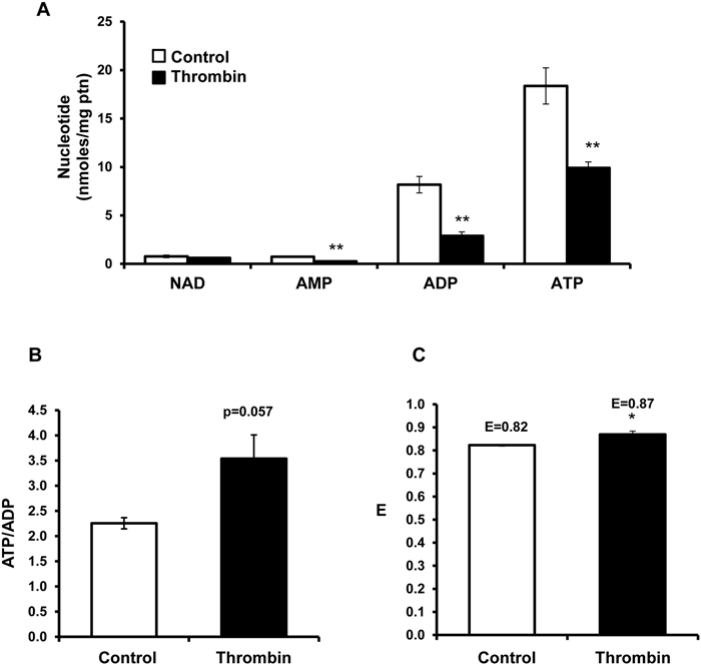
Changes in nucleotides after exposure of platelets to thrombin. Platelets (1 x 10^8^) were plated onto Cell-Tak coated 48 well plates, and exposed to either media or thrombin (0.5 U/ml) for 30 min and the samples were extracted to measure (A) NAD, AMP, ADP and ADP by HPLC. (B) The ATP/ADP ratio and (C) energy charge (ATP + 1/2ADP)/(ATP+ADP+AMP) was calculated. Data expressed as mean±SEM from one representative donor, n = 3 replicates per sample. *p<0.05, **p<0.01, different from control.

Platelets with and without thrombin were prepared and nucleotides measured by HPLC. On addition of thrombin (0.5 U/ml) to platelets, no significant change in NAD^+^ levels was observed (Fig 3A). In contrast, AMP, ADP and ATP levels significantly decreased, consistent with extrusion of nucleotides from the platelet upon activation. As an index of bioenergetic status, the ATP/ADP ratio was determined, and found to be increased on addition of thrombin consistent with an enhanced energetic status (Fig 3B). In addition, the energy charge (ATP + 1/2ADP)/(ATP+ADP+AMP) was also calculated [36]. The energy charge was significantly increased in the presence of thrombin (Fig 3C). Taken together with the data in Fig 1, showing an increased ATP linked respiration, and functional mitochondria (Fig 2), we conclude that mitochondrial integrity is maintained and contributes to the energetic demands imposed on the platelet by the addition of thrombin.

### Fatty acids as a substrate for platelet metabolism

**Fig 4:**
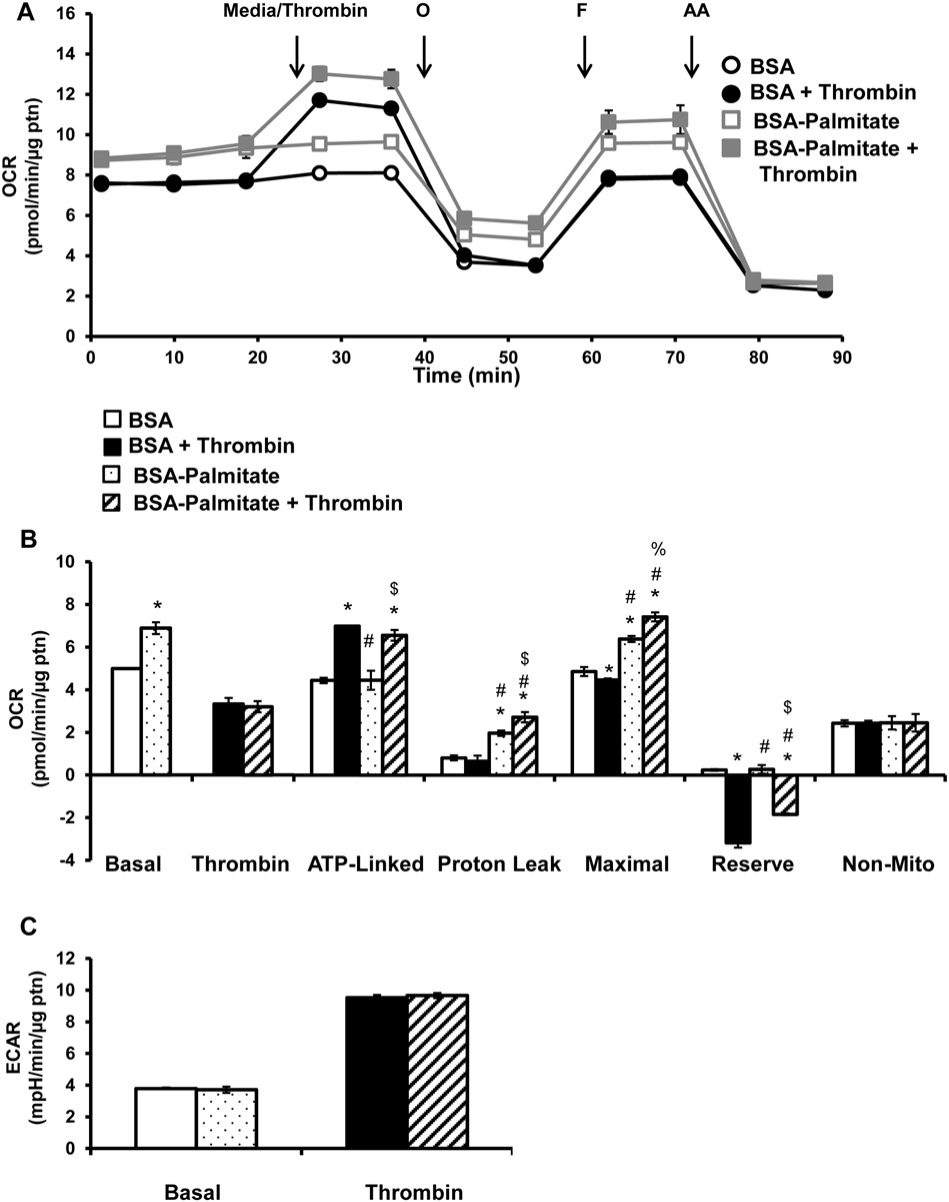
Effect of supplementation of BSA-palmitate on thrombin stimulated platelets. Platelets were plated on Cell-Tak coated XF96 plates, and pre-incubated with either BSA or BSA-palmitate (palmitate 200 μM) for 1h prior to bioenergetic measurements. (A) Basal OCR of platelets were measured prior to injection of thrombin (0.5 U/ml), followed by 1 μg/ml oligomycin (O), 0.6 μM FCCP (F) and 10 μM Antimycin A (AA). (B) Indices of mitochondrial function, basal, thrombin responsive, ATP-linked, proton leak, maximal, reserve capacity and non-mitochondrial OCR were calculated. (C) Basal and thrombin responsive ECAR were calculated from parallel ECAR measurements. Data expressed as mean±SEM from one representative donor, n = 3–5 replicates per sample. *p<0.01, different from control. #p<0.01, different from thrombin. %p<0.05 different from BSA-palmitate, $p<0.01 different from BSA-palmitate.

In order to assess the role of fatty acids as substrates for platelets, two approaches were taken. In the first, exogenous palmitate was provided to the platelets, since the XF DMEM media used in these experiments did not contain fatty acids. In the second approach, the effect of endogenous fatty acids on platelet respiration was tested using etomoxir, a CPT-1 inhibitor and TMZ, a 3-ketoacyl-CoA thiolase inhibitor, to inhibit fatty acid oxidation [37,38]. The bioenergetic assay was performed as shown in Fig 1C with pre-treatment of platelets with either BSA or BSA-palmitate (200 μM palmitate) for 1h. First, basal OCR was measured followed by the mitochondrial stress test. Addition of exogenous BSA-palmitate increased basal which could be accounted for by an increased proton leak, since ATP linked and non-mitochondrial OCR did not change. Maximal OCR was also increased compared to the BSA control, but without change in reserve capacity, due to the increased basal OCR (Fig 4A and 4B). Interestingly, BSA-palmitate did not affect the thrombin-dependent increase in OCR, indicating exogenous fatty acids do not contribute to thrombin stimulated increase in OCR (Fig 4A and 4B). Thrombin increased ATP-linked respiration as shown in Fig 1 which remained unchanged in the presence of BSA-palmitate (Fig 4B). Thrombin in the presence of BSA-palmitate further increased proton leak and maximal OCR when compared to BSA-palmitate alone (Fig 4B). Further, supplementation with BSA-palmitate was able to partially ameliorate the negative reserve capacity elicited by thrombin, consistent with a potential deficit in fatty acids to sustain maximal respiration (Fig 4B). Additionally, BSA-palmitate did not have any effect on either basal or thrombin stimulated ECAR (Fig 4C).

**Fig 5:**
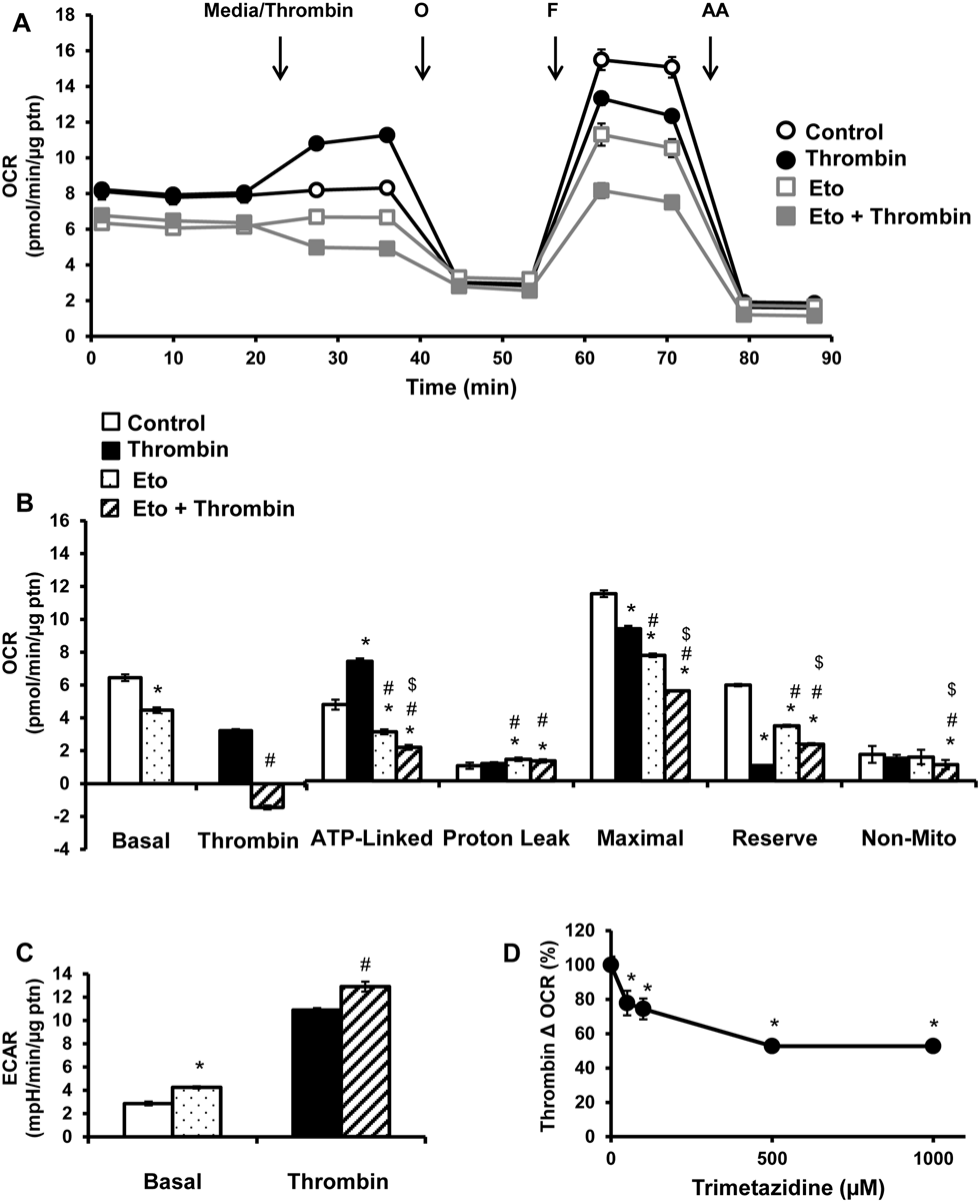
Effect of inhibiting mitochondrial fatty acid oxidation on thrombin stimulated platelets. Platelets were plated on Cell-Tak coated XF96 plates, and pre-treated with etomoxir (25 μM) for 1h prior to bioenergetic measurements. (A) Basal OCR of platelets were measured prior to injection of thrombin (0.5 U/ml), followed by 1 μg/ml oligomycin (O), 0.6 μM FCCP (F) and 10 μM antimycin A (AA). (B) Indices of mitochondrial function, basal, thrombin responsive, ATP-linked, proton leak, maximal, reserve capacity and non-mitochondrial OCR were calculated. (C) Basal and thrombin responsive ECAR were calculated from parallel ECAR measurements. Platelets were pre-treated with TMZ (0–1000 μM) for 3h before bioenergetic assay. (D) Change in OCR after thrombin injection presented as a percentage of control. Data expressed as mean±SEM from one representative donor, n = 3–5 replicates per sample. *p<0.01, different from control. #p<0.01, different from thrombin. $p<0.01 different from etomoxir.

Next, the role of endogenous fatty acids in platelet bioenergetics was tested by pre-treatment with etomoxir (25 μM) and then basal respiration was measured followed by the mitochondrial stress test. Inhibition of endogenous fatty acid oxidation decreased basal, ATP-linked, maximal OCR and reserve capacity (Fig 5A and 5B), indicating that resting platelets utilize fatty acids to produce ATP. Etomoxir suppressed the thrombin mediated stimulation of OCR (Fig 5A and 5B). While thrombin increased ATP-linked OCR, etomoxir decreased both unstimulated and thrombin stimulated ATP-linked respiration (Fig 5A and 5B), and led to an increase in proton leak (Fig 5A and 5B). Thrombin injection led to a decrease in maximal respiration and reserve capacity, which was further decreased in the presence of etomoxir (Fig 5A and 5B). The combination of etomoxir and thrombin decreased non-mitochondrial OCR (Fig 5A and 5B).

The presence of etomoxir led to an increase in basal ECAR compared to control, possibly as a compensatory increase in glycolysis, due to the decreased mitochondrial ATP production (Fig 5C). Thrombin alone increased ECAR, (see also Fig 1), both etomoxir and thrombin together further increased ECAR, suggesting that glycolysis is capable of compensating for the loss of mitochondrial-dependent fatty acid oxidation (Fig 5C).

To confirm the effect of endogenous fatty acid oxidation on platelet metabolism, trimetazidine (TMZ) was used. Platelets were pre-incubated with TMZ (0–1000 μM) for 3h after which the mitochondrial stress test was performed. TMZ at the highest concentration which does not affect platelet aggregation (500 μM), caused an approximately 50% decrease in thrombin stimulated OCR (Fig 5D), again indicating that endogenous fatty acids are a source of fuel to support platelet metabolism in the presence of thrombin. It has been previously reported that TMZ at doses of 1000 μM and higher, inhibit platelet aggregation through the blunting of calcium influx caused by thrombin, an event required for aggregation to occur [39].

### Gln as a substrate for platelet metabolism

**Fig 6:**
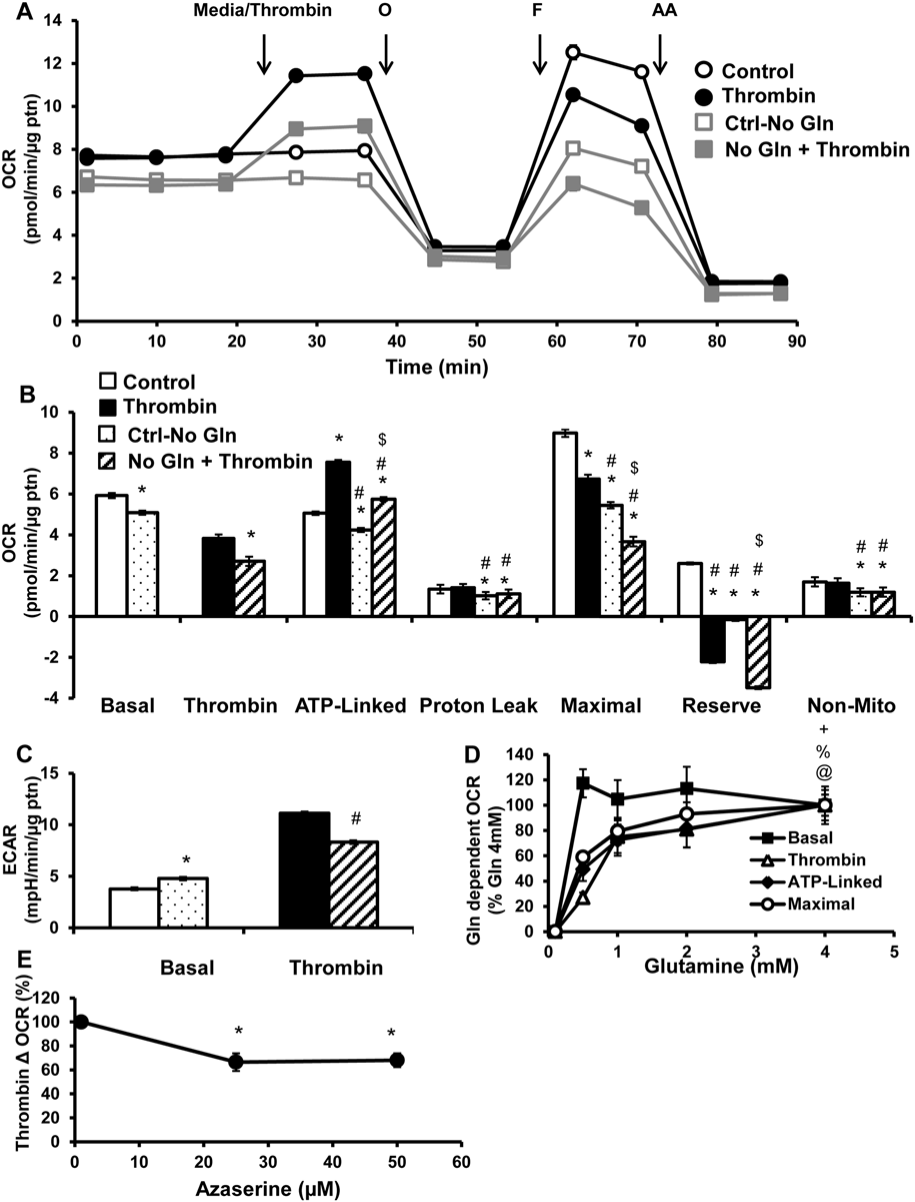
Effect of Gln on thrombin stimulated platelets. Platelets were plated on XF96 plates Cell-Tak coated, and incubated in either regular XF DMEM media or media without Gln for 1h, before bioenergetic measurements. (A) Basal OCR of platelets were measured ahead of thrombin injection (0.5 U/ml), followed by 1 μg/ml oligomycin (O), 0.6 μM FCCP (F) and 10 μM antimycin A (AA). (B) Indices of mitochondrial function, basal, thrombin responsive, ATP-linked, proton leak, maximal, reserve capacity and non-mitochondrial OCR were calculated. (C) Basal and thrombin responsive ECAR were calculated from parallel ECAR measurements. Bioenergetic assays were performed in XF DMEM media containing different concentrations of Gln (0–4 mM). (D) Basal OCR prior to thrombin injection, thrombin linked, ATP linked and maximal OCR after thrombin injection presented as a percentage of highest concentration of Gln (4 mM) after subtraction of no Gln OCR. Platelets were pre-treated with Aza (0–50 μM) for 3h before bioenergetic assay. (E) Change in OCR after thrombin injection presented as a percentage of control. Data expressed as mean±SEM from one representative one donor, n = 3–5 replicates per sample. *p<0.01, different from control. #p<0.01, different from thrombin. $p<0.01 different from Ctrl-no glut. @p<0.01, thrombin linked OCR different from Gln (500 μM). %p<0.01, ATP-linked OCR different from Gln (500 μM). +p<0.01, maximal OCR different from Gln (500 μM).

Gln contributes to ATP production through the process of glutaminolysis, by providing substrates for the TCA cycle. In this experiment the effect of Gln depletion on platelet bioenergetics was assessed by removing it from the XF DMEM assay media, for 1h before the mitochondrial stress test. This led to a decrease in basal, ATP-linked, proton leak, maximal, reserve capacity and non-mitochondrial OCR (Fig 6A and 6B). Without Gln, thrombin stimulated OCR is also significantly suppressed (Fig 6A and 6B). There was no additional effect of thrombin and Gln depletion on ATP-linked, proton leak, maximal and non-mitochondrial OCR (Fig 6A and 6B). However, the reserve capacity in the thrombin and no Gln group was further decreased compared to the Gln-depleted group (Fig 6B). Similar to the etomoxir treatment, Gln depletion caused a compensatory increase in basal ECAR, but in contrast to etomoxir, ECAR was not further increased by thrombin, compared to thrombin alone group, with Gln depletion (Fig 6C).

Since the concentration of Gln used in the XF DMEM media is 5–8 fold higher than reported in the plasma, a concentration dependence of Gln (0–4 mM) on platelet bioenergetics was established. In this analysis we have set the highest Gln (4mM)-dependent change in OCR as 100%, for each bioenergetic parameter to allow comparisons (Fig 6D). Basal respiration (before thrombin addition) was maximal at the lowest concentration of Gln tested (500 μM). In contrast, the other parameters showed concentration dependence with Gln. Importantly, the thrombin-dependent OCR exhibited an EC_50_ (half maximal effective concentration) of approximately 1500 μM. Whereas ATP-linked and maximal or FCCP-dependent OCR after thrombin addition, were slightly less sensitive, with an EC_50_ of 1000 and 750 μM respectively. As a further test of the effects of Gln on platelet metabolism, azaserine (Aza), a Gln analog that inhibits glutaminase and glutamine amidotransferases was used [40]. Platelets were pre-treated with Aza (0–50 μM) for 3h, after which thrombin stimulated OCR was measured. Aza (25 and 50 μM) inhibited thrombin stimulated OCR by approximately 40%, similar to Gln depletion (Fig 6E), confirming that Gln plays a role in supporting mitochondrial respiration after thrombin addition.

### Glucose as a substrate for platelet metabolism

**Fig 7:**
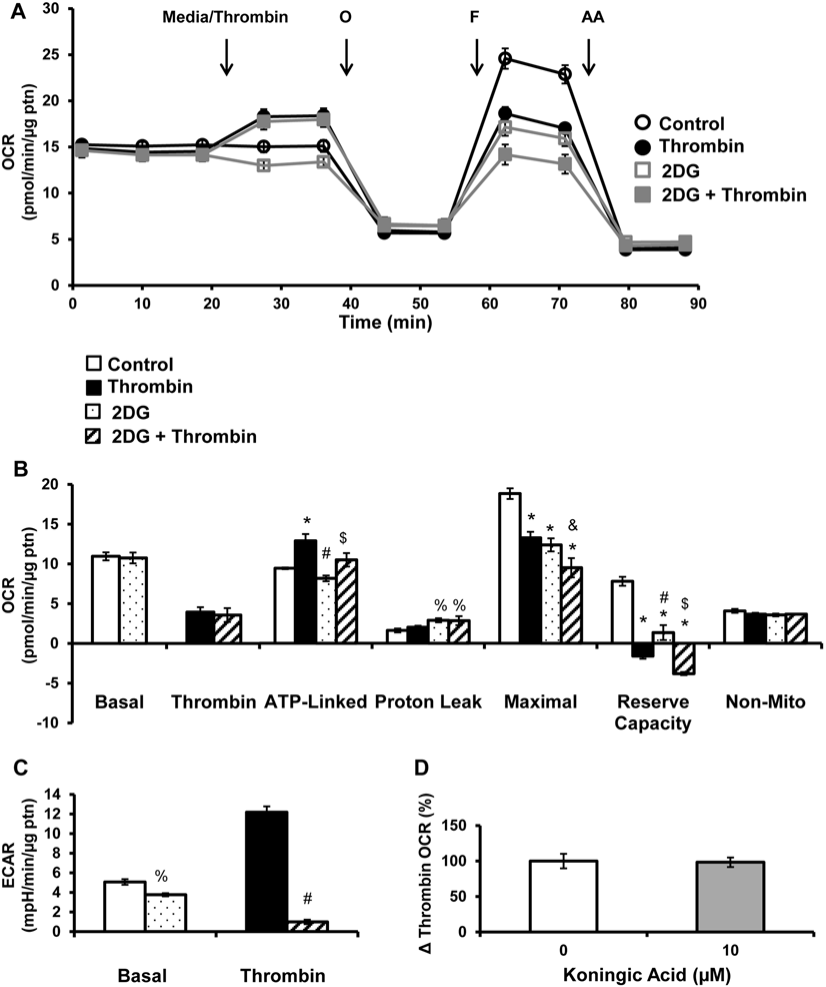
Effect of inhibiting glycolysis on thrombin stimulated platelets. Platelets were plated on Cell-Tak coated XF96 plates, and pre-treated with 2DG (120 mM) for 1h before the bioenergetic assay. (A) Basal OCR of platelets were measured prior to thrombin injection (0.5 U/ml), followed by 1 μg/ml oligomycin (O), 0.6 μM FCCP (F) and 10 μM antimycin A (AA). (B) Indices of mitochondrial function, basal, thrombin responsive, ATP-linked, proton leak, maximal, reserve capacity and non-mitochondrial OCR were calculated. (C) Basal and thrombin responsive ECAR were calculated from parallel ECAR measurements. Platelets were pre-treated with koningic acid (10 μM) for 1h prior to the bioenergetic assay. (E) Change in OCR after thrombin injection presented as a percentage of control. Data expressed as mean±SEM from one representative one donor, n = 3–5 replicates per sample. *p<0.01, different from control. %p<0.05, different from control. #p<0.01, different from thrombin. &p<0.05, different from thrombin. $p<0.01 different from 2DG.

In the next series of experiments, glucose oxidation was examined using the inhibitor of hexokinase, 2-deoxy-D-glucose (2DG) and koningic acid, an inhibitor of glyceraldehyde-3-phosphate dehydrogenase. Platelets were pre-treated with 2DG (120 mM) for 1h, prior to bioenergetic measurements as described above. 2DG alone did not significantly change basal, ATP-linked or non-mitochondrial OCR, but decreased maximal OCR, reserve capacity, and increased proton leak (Fig 7A and 7B). 2DG did not significantly affect the thrombin induced increase in OCR or the thrombin-dependent increase in ATP linked respiration (Fig 7A and 7B). The combined treatment of 2DG and thrombin decreased both maximal and reserve capacity compared to 2DG alone (Fig 7A and 7B). As expected, 2DG treatment decreased basal ECAR and essentially abolished the thrombin mediated increase in ECAR (Fig 7C). Platelets pre-treated with koningic acid (10 μM) for 1h, also had no effect on thrombin stimulated OCR.

### Effect of metabolic inhibitors on platelet aggregation

**Fig 8:**
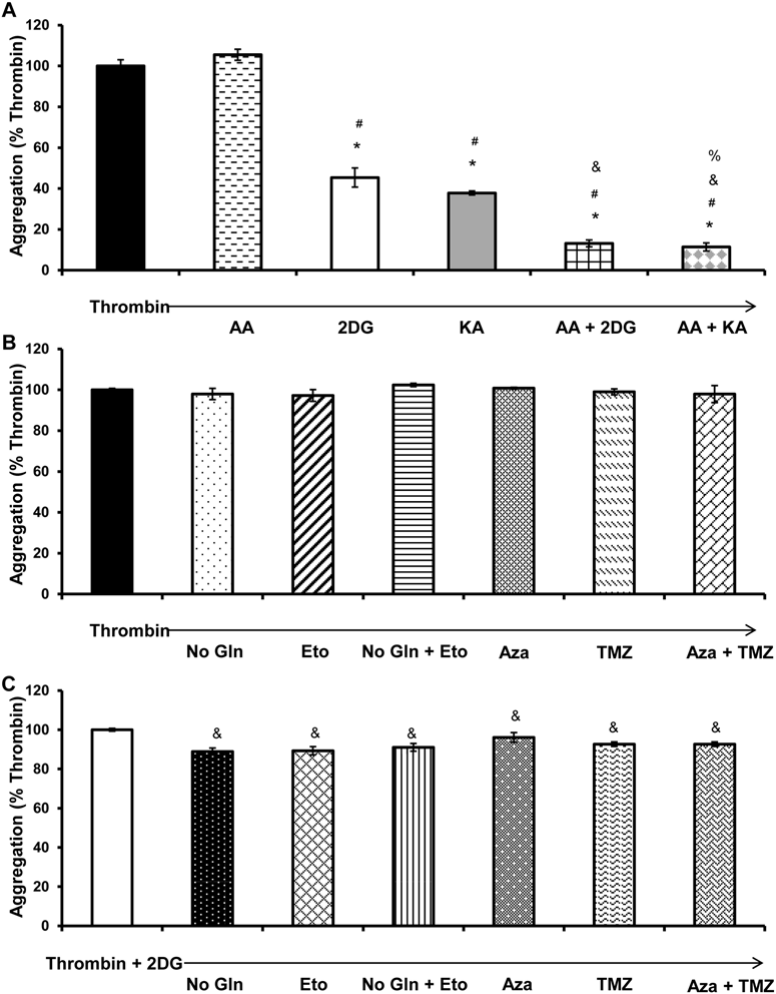
Aggregation of platelets stimulated with thrombin in the presence of metabolic inhibitors. Platelets (40 x 10^6^) in suspension were plated onto 96 well plate reader plates, and change in light transmittance following thrombin (0.5 U/ml) addition was measured in platelets subjected to either 1h or 3h treatments consistent with bioenergetic assays, under the following conditions (A) antimycin A (AA, 10 μM), 2DG (120 mM), koningic acid (KA, 10 μM), AA+2DG together and KA+2DG together (B) presence and absence of Gln in media, etomoxir (25 μM) alone and in combination and TMZ (500 μM) and Aza (25 μM) alone and in combination (C) depletion of Gln, etomoxir, TMZ, and Aza together with 2DG. Extent of aggregation is expressed as a percentage of thrombin 0.5 U/ml. Data expressed as mean±SEM from one representative donor, n = 3 replicates per sample. *p<0.01, different from thrombin. #p<0.01, different from thrombin + AA. $p<0.01, different from thrombin + 2DG.

In order to assess how metabolic inhibitors and substrate availability affect platelet aggregation, the effects of antimycin A (10 μM), 2DG (120 mM), koningic acid (10 μM), etomoxir (25 μM), TMZ (500 μM), Gln depletion, Aza (25 μM) either alone or in combination were determined, following the addition of thrombin (0.5 U/ml) to initiate aggregation. Addition of antimycin A alone had no effect on aggregation (Fig 8A), which we ascribe to the compensatory increase in glycolysis which occurs on addition of the mitochondrial inhibitor. Whereas, 2DG and koningic acid alone inhibited aggregation by over 50% (Fig 8A). Antimycin A and 2DG in combination or antimycin A and koningic acid, synergistically inhibited platelet aggregation (Fig 8A). Etomoxir, Gln-depletion, alone or in combination, do not affect the ability of platelets to aggregate (Fig 8B). Similarly, TMZ and Aza, alone or in combination do not affect platelet aggregation (Fig 8B). Interestingly, etomoxir or Gln, in combination with 2DG, decreased platelet aggregation by a further 10% compared to 2DG alone (Fig 8C). A similar effect was observed with TMZ and Aza, in combination with the glycolytic inhibitor 2DG (Fig 8C).

## Discussion

Major thrombotic and hemorrhagic events are associated with changes in platelet metabolism and function including aggregation [6,8–10,41–44]. Understanding these metabolic changes is important in designing new therapeutic approaches to modulate platelet function, and assessing the impact of current interventions for cancer or other diseases on platelet bioenergetic capacity. Although it is clear that both glycolysis and oxidative phosphorylation are active metabolic pathways in platelets, the metabolic plasticity in the use of oxidative substrates for platelet aggregation remains unexplored [6,11–15]. The focus of this study is to elucidate how fatty acids and Gln interact with glucose in regulating the metabolic changes that accompany thrombin mediated platelet aggregation. The advantage of the approach taken here is that the changes in platelet metabolism can be followed over time, and mitochondrial function can be determined in intact and permeabilized platelets, thereby avoiding artifacts introduced by disruptive isolation techniques.

Using platelets isolated from healthy human volunteers, we simultaneously measured mitochondrial function and glycolysis after exposing platelets to thrombin and found a rapid stimulation of both metabolic pathways (Fig 1). Interestingly, thrombin increases ATP-linked respiration which is direct evidence for the contribution of the mitochondrion to the energetic demand associated with thrombin stimulated aggregation. Other oxygen consuming processes such as the cyclooxygenases were probably below the limit of detection. It has been suggested that high levels of thrombin can lead to opening of the mitochondrial permeability transition pore during platelet aggregation, leading to decreased mitochondrial membrane potential and reduced ATP output [45,46]. However, we have shown that over the first 30 min after thrombin exposure there is no change in proton leak (Fig 1C and 1D). This indicates that the mitochondrial permeability transition pore has not opened under these conditions, since it would increase proton leak and decrease ATP linked respiration. Further, measurement of mitochondrial function after thrombin exposure revealed only minor changes in RCR and oxidation of complex I and II linked substrates, which suggests that a major mitochondrial dysfunction is not normally associated with the early stages of platelet aggregation (Fig 2). There is a decrease in total ATP and ADP levels in thrombin treated platelets indicative of granule release (Fig 3A), but bioenergetic parameters such as the energy charge and ATP/ADP ratio are maintained indicating that the energy demand of the platelet can be met under these conditions of increased demand (Fig 3).

It is not known which substrates support the rapid and sustained stimulation of mitochondrial respiration and glycolysis upon addition of thrombin in platelets. The XF DMEM media used in these experiments did not contain free fatty acids. The source of fatty acids for oxidative phosphorylation is likely derived from endogenous free fatty acids and triglycerides stores that release fatty acids upon lipase activity [47,48]. It is important to note that fatty acid oxidation also occurs in the peroxisomes and with enzymes such as cyclooxygenase [49,50]. However, the oxygen consumption rate reported here can largely be ascribed to mitochondrial oxidative phosphorylation, since it is inhibited by antimycin A. Exogenous supplementation with the fatty acid palmitate, increased basal and maximal respiration, indicating that platelets are able to transport extracellular fatty acids and utilize them for respiration (Fig 4). Fatty acid supplementation also increases proton leak, which has been described previously [51–53]. However, supplementation of fatty acids do not change thrombin stimulated respiration, indicating that the platelets are not relying on exogenous fatty acids to support the metabolic requirements for thrombin stimulation (Fig 4). To elucidate the contribution of endogenous stores of fatty acids to platelet bioenergetics, using etomoxir, an inhibitor of CPT-1, we found that fatty acid oxidation is making a major contribution to basal respiration and is required for the thrombin stimulated oxygen consumption (Fig 5B). Using TMZ which inhibits 3-ketoacyl-CoA thiolase, we similarly found that fatty acid oxidation is required for thrombin linked increase in OCR, albeit to a lower extent than etomoxir (Fig 5D). The reasons for this difference are not clear, and could be due to an off-target effect of etomoxir or compensation in the presence of TMZ.

Gln depletion from the media or inhibition of glutaminase with Aza resulted in a small but significant effect on platelet bioenergetics (Fig 6A and 6E). Further, a Gln concentration dependent effect was found on bioenergetic parameters in the presence of thrombin (Fig 6D). In the absence of thrombin, bioenergetic parameters showed minimal dependence on Gln, with the exception of maximal respiration which exhibited an EC_50_ of 367 μM (data not shown). Since the physiological concentrations of Gln in plasma of healthy subjects are 500–750 μM [54], then Gln could become limiting for platelet bioenergetics. This may be important under conditions of Gln deficiency, which have been reported to occur after infections or injury [55]. Interestingly, although glucose oxidation is substantially increased on addition of thrombin it is not making a significant contribution to the thrombin-dependent stimulation of mitochondrial function (Fig 7).

An interesting feature of these studies is the plasticity of platelet metabolism. For example, Gln depletion and inhibition of fatty acid oxidation both caused a compensatory increase in basal ECAR (Fig 5C and 6C). The acidification of the media, measured as an increase in ECAR, is potentially derived from several sources including proton production during glycolysis and from mitochondrial respiration derived CO_2_ that forms carbonic acid [56]. Using 2DG, an inhibitor of hexokinase, the contribution of glycolysis to ECAR was confirmed, since in resting platelets, 36% of the ECAR and in thrombin stimulated platelets 90% of the ECAR, was inhibitable by 2DG (Fig 7C) Further, inhibition of mitochondrial fatty acid oxidation also causes a compensatory increase in thrombin stimulated glycolysis, but this effect is not observed in the absence of Gln (Fig 5C and 6C). As expected, inhibiting glycolysis decreased basal and thrombin stimulated ECAR (Fig 7C).

The mitochondrial metabolism inhibitors antimycin A, etomoxir, TMZ, Gln depletion, Aza alone or in combination do not prevent platelet aggregation (Fig 8B). We ascribe this effect to a switch to glycolytic metabolism, to compensate for the decrease in oxidative phosphorylation. However, it appears that platelets have a requirement for glucose oxidation which cannot be compensated for by mitochondrial function, since hexokinase inhibition with 2DG and glyceraldehyde-3-phosphate dehydrogenase inhibition with koningic acid, significantly inhibit platelet aggregation (Fig 8A). The mechanisms underlying these effects are not clear, since both compounds act on different steps in glycolysis, it is likely that the pentose phosphate pathway, which is inhibited by 2DG but not koningic acid, is not playing a major role regulating the energetics of platelets. The combined effects of 2DG or koningic and antimycin are synergistic in the inhibition of platelet aggregation consistent with published studies (Fig 8A) [10]. In contrast, combining inhibition of glycolysis, with etomoxir and/or Gln depletion, or TMZ and/or Aza, decreases aggregation by a further 10%, compared to glycolytic inhibition alone, providing further evidence for multiple oxidative substrate usage in platelet metabolism (Fig 8C).

In summary, we have used a novel approach to measure the oxidative and glycolytic changes that occur with thrombin stimulation of platelets. Here, we show that fatty acids and Gln are required for the rapid thrombin-dependent stimulation of oxidative phosphorylation but not for thrombin-dependent aggregation. Thrombin-dependent activation of platelets initiates a metabolic reprogramming of the platelet towards aerobic glycolysis, increased fatty acid oxidation and glutaminolysis, which is fully capable of meeting the energetic demand imposed by aggregation and underlines the metabolic plasticity of the platelet.
